# Improved Release of Monosaccharides and Ferulic Acid Using Enzyme Blends From *Aspergillus Niger and Eupenicillium Parvum*

**DOI:** 10.3389/fbioe.2021.814246

**Published:** 2022-01-27

**Authors:** Zhenghui Liu, Enze Shi, Feng Ma, Xin Zhou, Kankan Jiang

**Affiliations:** ^1^ School of Basic Medical Sciences and Forensic Medicine, Hangzhou Medical College, Hangzhou, China; ^2^ Jiangsu Co-innovation Center of Efficient Processing and Utilization of Forest Resources, College of Chemical Engineering, Nanjing Forestry University, Nanjing, China

**Keywords:** wheat bran autohydrolysis residue, enzymatic hydrolysis, monosaccharide, ferulic acid, enzyme blend

## Abstract

Supplementing commercial xylanase and cellulase with selected debranching enzymes only resulted in slight enhancement of the enzymatic hydrolysis of wheat bran autohydrolysis residues (WBAR) which was obtained at 160°C over a 30-min period of autohdyrolysis, while a blend of enzymes from *Aspergillus niger* and *Eupenicillium parvum* achieved synergistic efficacy in this context. Using an equal mixture blend of these enzymes at a 0.5% (w/w) enzyme loading dosage with the addition of ferulic acid esterase (1 U/g substrate), the obtained hydrolysis yields were desirable, including 84.98% of glucose, 84.74% of xylose, 80.24% of arabinose, and 80.86% of ferulic acid. Following further separation using an HP-20 resin, the final ferulic acid recovery levels were as high as 62.5% of the esterified ferulic acid present within the initial WBAR input. Together, these data suggest that a combination of autohydrolysis and enzymatic hydrolysis using crude enzyme blends can efficiently achieve wheat bran enzymatic saccharification and associated ferulic acid release.

## Introduction

Wheat bran, which accounts for 25% of the weight of wheat grains, is a byproduct of the wheat production process. Approximately 150 million tons of wheat bran are generated globally each year, and this bran is most commonly utilized as a feed for livestock (Si et al., 2020). However, bran-based feeds are of low commercial value, leading to efforts in the wheat industry to convert this byproduct into a higher value commodity. Wheat bran is composed primarily of arabinoxylan (38–55% of dry mass), cellulose (16–30%), and esterified ferulic acid ( ∼ 1%). Ferulic acid (FA) offers value as an antimicrobial compound with antioxidant properties, and it can additionally serve as a preservative for food products (Li et al., 2021; Hu et al., 2022). Moreover, it can be converted into vanillin for use as a flavoring agent in the food and perfume industries (Ferri et al., 2020). Wheat bran thus represents an ideal low-cost, abundant, organic source of natural FA production and a valuable source for other biochemical compounds of potential commercial value.

Most studies to date have primarily focused on preparing and processing wheat bran-derived xylooligosaccharides (Mathew et al., 2017; Wu et al., 2017; Bhattacharya et al., 2020; Sonkar et al., 2021) or arabinoxylan (Aguedo et al., 2014; Sánchez-Bastardo et al., 2017; Kaur et al., 2021). Other researchers have sought to improve wheat bran utilization efficiency by developing approaches to generating fermentable sugars as products of wheat bran decomposition, providing an efficient source for the precursors necessary for bioethanol preparation. Enzymatic saccharification can maintain the production of these fermentable sugars without favoring the concomitant production of inhibitory compounds (Huang et al., 2022). However, achieving complete enzyme-mediated hydrolysis of raw wheat bran remains challenging owing to the complexity and heterogeneity of arabinoxylan. Wheat bran arabinoxylan exhibits a high degree of substitution, with a β-1,4-linked xylopyranose backbone linked to α-L-arabinofuranose units that can either be unsubstituted or exhibit xylose C(O)-2 and/or C(O)-3 substitutions (Gullón et al., 2014). Moreover, these molecules can exhibit α-1,2-linked glucuronic, acetyl, D-galactopyranosyl or methyl-glucuronic acid residue substituents, and ferulic acid can be esterified to arabinose units at the O-5 position (Mathew and Abraham, 2004; Ma et al., 2012; Peng et al., 2012). Additionally, ferulic acid can form dehydrodimers that can facilitate arabinoxylan polymer cross-linking (Mandalari et al., 2005). Owing to these complexities, highly efficient enzymatic cocktails are essential to fully unlock the commercial and biological potential of wheat bran polysaccharides. On the other hand, in prior studies, the underlying utility of wheat bran-derived ferulic acid generated in the context of enzymatic hydrolysis has largely been overlooked, highlighting an untapped resource that has the potential to be enriched from prepared enzymatic hydrolysates.

To alter the chemical and microscopic structural properties of wheat bran and to increase its amenability to subsequent enzymatic hydrolysis, bran initially undergoes pretreatment processing. Prior studies focused on bioethanol production have utilized H_2_SO_4_, H_3_PO_4_, or other acids to liberate sugars from wheat bran (Palmarola-Adrados et al., 2005; Cripwell et al., 2015; Nair et al., 2015). While this resulted in acceptable rates of sugar recovery, such harsh acidic pretreatment ultimately results in the degradation of a portion of the sugar molecules. In contrast, hydrothermal autohydrolysis-based pretreatment efforts are cost-effective, straightforward, and do not incur significant environmental harm (Batalha et al., 2015; Khalili and Amiri, 2020; Wang et al., 2021). Mild heat-based autohydrolysis treatment can facilitate partial arabinoxylan depolymerization and debranching, breaking down complex hemicellulose molecular networks within wheat bran. However, the full degradation of wheat bran substrates necessitates the use of a complex enzymatic mixture including both cellulolytic and hemicellulolytic enzymes. Owing to enzymatic synergy such that the products of a given reaction can serve as substrates for a different enzyme, crude enzyme extract blends with extensive enzyme activities can enhance lignocellulose hydrolytic efficiency (Saini et al., 2016). For example, α-L-arabinofuranosidases are unable to liberate L-arabinofuranosyl residue (Ara*f*) that are esterified with phenolic acids (Biely et al., 2016). Therefore, the prior action of ferulic acid esterase (FAE) to liberate *trans*-ferulic acid from 5-O-feruloylated Ara*f* is essential for the subsequent action of α-L-arabinofuranosidases. Besides, WB xylan backbone is highly substituted with arabinofuranosyl substituents singly attached to C(O)-2/C(O)-3, or doubly linked to C(O)-2,3 of the xylose residues (Sørensen et al., 2007a; Sakamoto et al., 2011). These arabinose substitutions partly impede the action of endo-xylanase by causing steric hindrance for accessing the β-1,4-bonds in xylan, resulting in the limited release of xylobiose, xylotriose and other short-chain xylooligosaccharides from partially shaved xylan backbone (Sørensen et al., 2005; Sørensen et al., 2007a). In other words, the depolymerization of xylan backbone by endo-xylanase is depended on the removal of the arabinofuranosyl residues from arabinoxylan by arabinofuranosidases catalysis, and this depolymerization also makes β-xylosidase perform better on the catalysis release of xylose from produced xylobiose, xylotriose, and short-chain xylooligosaccharides (Sørensen et al., 2005; Sørensen et al., 2007b). As such, wheat bran autohydrolysis residue (WBAR) derived from the pretreatment of de-starched wheat bran using hot water was subjected to further enzymatic hydrolysis in order to facilitate monosaccharide and FA recovery through synergistic enzymatic activity using a blend of crude enzymatic extracts derived from *Aspergillus niger* and *Eupenicillium parvum*. This strategy offers potential value as a reliable approach to biomass hydrolysis for the preparation of fermentable sugars and natural FA. Many different fungal strains such as those belonging to *Aspergillus*, *Trichoderma* as well as *Penicillium*, and their combinations have been used to prepare synergistic cellulase blends either by co-culture or by mixing of broths in the past. However, as far as our knowledge is concerned, there are fewer reports on the synergistic crude enzyme preparations from *Eupenicillium* and *Aspergillus.*

## Materials and Methods

### Materials

Wheat bran was obtained from Nanyang, Henan Province, China, and was treated with papain and amylase (Imperial Jade Biotechnology Co., Ltd., Ningxia, China) based on a modified version of the protocol developed by Rose and Inglett to yield de-starched WB (DSWB) (Rose and Inglett, 2010). One hundred grams of wheat bran were treated with 0.3% amylase enzyme solution at 65°C for 30 min, and then incubated at 55°C for 30 min after addition of 0.3% (w/v) papain enzyme, followed by heat deactivation at 99°C for 20 min and extensive washing with distilled water to remove starch. Commercial cellulase, β-glucosidase, and xylanase were obtained from Sigma Aldrich (MO, USA). Arabinofuranosidase (AF) and acetyl xylan esterase (AXE) were from Megazyme (Bray, Ireland). FA esterase (FAE) derived from *Myceliopthora thermophila* (ATCC 42464) that had been recombinantly expressed in *Pichia pastoris* strain X33 was provided as a kind gift by Prof. Ding of Nanjing Forestry University, who also provided the hemicellulolytic enzyme-producing *E. parvum* 4–14 strain. *E. parvum* 4–14 was isolated from soil (Nanjing, China) and deposited in the China Center for Type Culture Collection (CCTCC) (Long et al., 2016). All other chemicals used herein were of analytical grade and were from Sinopharm Chemical Reagent Co., Ltd. (Shanghai, China). *A. niger* TRIIM 3.00944 was from Tianjin Institute of Industrial Microorganism in China.

### Solid-State Fermentation

Crude enzymatic preparations were prepared *via* SSF from *A. niger* and *E. parvum* strains grown in modified Mandel’s medium in which wheat bran served as a carbon source as per the methods described previously by Long et al. (Long et al., 2016). In a 250 ml Erlenmeyer flask, for every 1.5 g of delignified wheat straw (1–2 mm), 1.5 g of DSWB was mixed with 5 ml of 10 × Mandel’s medium without any other carbon source, followed by sterilization at 121°C for 20 min. A fungal block derived from a PDA slant was then inoculated into a 250 ml Erlenmeyer flask containing 50 ml of liquid medium, followed by culture for 7 days at 37°C with constant agitation (200 rpm). Then, 2 ml of the prepared fungal culture was used to inoculate the Erlenmeyer flask containing SSF medium prepared above, followed by fermentation for 10 days at 37°C with 70% humidity. Following the completion of this fermentation step, flasks were mixed well with 25 ml of sterilized H_2_O supplemented with 0.1% (v/v) Tween-80, shaken (120 rpm) for 2 h at 28°C, and centrifuged for 10 min at 7,000 × g. Supernatants were then transferred to fresh tubes for enzymatic analyses. Tetracycline was added to crude enzyme preparations (0.05%, w/v), followed by storage at 4°C.

### WBAR Preparation

A stainless steel batch reactor (model YRG2-10 × 1.25 L, ZhengJie Technology and Development Co., Ltd., Nanjing, China) was utilized for autohydrolysis. Briefly, DSWB (50 g) was added to the reactor and mixed with ultrapure water (500 ml) followed by immersion in an oil bath. Autohydrolysis was then conducted for 30 min at 160°C, not including periods for heating and cooling. Following the completion of autohydrolysis, reactors were immersed in cool water to lower the reaction system temperature to the ambient temperature. Solid residues were then washed repeatedly using tap water, collected on a filtration cloth, and dried under vacuum for 24 h at 40°C. Samples were then sealed in zipper-locked bags prior to subsequent composition analyses and enzymatic hydrolysis.

### WBAR Hydrolysis Using Commercial Enzymes or Crude Enzyme Blends

WBAR was used as a substrate for enzymatic hydrolysis performed in 50 ml conical flasks at a substrate loading of 5% (w/v, 5 ml total volume) at 50°C using 50 mM sodium citrate buffer (pH 5.0). Flasks were constantly agitated (150 rpm) for 72 h, with added enzymes including 15 CBU β-glucosidase (Novozyme 188, 269 CBU/g), 15 FPU cellulase (Sigma C2730, 117 FPU/g), and 200 U xylanase (Sigma X2629, 7700 U/g) per gram of dry biomass. Tetracycline (0.05%, w/v) was added to prevent bacterial contamination. Reactions were terminated *via* transferring the flasks into boiling water for 10 min and then clarifying hydrolysates *via* centrifugation. Supernatant glucose, xylose, and arabinose levels were assessed *via* HPLC. Analyses were repeated in duplicate. Glucose and xylose yields were determined with the following equations:

$$\text{Glucose yield }\left(\text{\%}\right)\;=\;\frac{100\%\;\times \;\left(0.9\;\times \text{ glucose released following enzymatic hydrolysis}\right)}{\text{amount of cellulose in WBAR}}$$ (1)

$$\text{Xylose yield }\left(\text{\%}\right)=\;\frac{100\%\;\times \;\left(0.88\;\times \text{ xylose released following enzymatic hydrolysis}\right)}{\text{amount of xylan in WBAR}.}$$ (2)

The conversion factor for dehydration on polymerization to cellulose was 162/180 (0.9) for glucose; to xylan and arabinan, it was 132/150 (0.88) for xylose and arabinose, respectively.

In experiments in which debranching enzymes were added, experimental protocols were as above with the addition of 1 U of AF (Megazeme, E-ABFAN), AXE (Megazeme, E-AXEAO), and FAE complemented with 15 FPU of cellulase, 15 CBU of β-glucosidase, and 200 U of xylanase. FA oxidation was prevented *via* the addition of sodium hydrogen sulfite (100 mg/L). Glucose and xylose yields were calculated as above, while arabinose, FA, and acetic acid yields were calculated with the following equations:

$$\text{Arabinose yield }\left(\text{\%}\right)\;=\;\frac{100\%\;\times \;\left(0.88\;\times \text{ arabinose released following enzymatic hydrolysis}\right)}{\text{amount of arabinan in WBAR}}$$ (3)

$$\text{Ferulic acid yield }\left(\text{\%}\right)\;=\;\frac{100\%\;\times \;\left(\text{ferulic acid released following enzymatic hydrolysis}\right)}{\text{amount of esterified ferulic acid in WBAR}}$$ (4)

$$\text{Acetic acid yield }\left(\text{\%}\right)\;=\;\frac{100\%\;\times \;\left(\text{acetic acid released following enzymatic hydrolysis}\right)}{\text{amount of acetic acid in WBAR}}$$ (5)

WBAR enzymatic hydrolysis using crude blends of enzymes derived from *E. parvum* and *A. niger* was conducted as above at a range of experimentally appropriate enzyme doses. Monosaccharide and FA hydrolysis yields were calculated using Equations 1–4.

### Carbohydrate, Ferulic Acid, and Acetic Acid Analyses

A two-step sulfuric acid-based hydrolysis approach was used to measure xylan, cellulose, arabinan, and other structural carbohydrates derived from WBAR (Sluiter et al., 2005). An HPLC approach was utilized to measure levels of glucose, xylose, and arabinose in the acid hydrolysate samples by using an Agilent 1,100 (USA) instrument with a Bio-Rad Aminex HPX-87H column (300 mm × 7.8 mm; USA) and a refractive index detector. This analytical column was used at a constant 55°C temperature with a mobile phase composed of H_2_SO_4_ (5 mM) and a constant 0.6 ml/min flow rate. The conversion factor for dehydration on polymerization to cellulose was 162/180 (0.9) for glucose; to xylan and arabinan, it was 132/150 (0.88) for xylose and arabinose. All analyses were conducted in triplicate.

Levels of acetic acid and monosaccharides present within enzymatic hydrolysates were measured *via* HPLC as above. Esterified FA levels in WBAR were measured following NaOH saponification as in a prior report (Jiang et al., 2016). Levels of free FA were assessed *via* HPLC (Agilent Technologies 1,260 Infinity) with a ZORBAX Eclipse Plus C18 column (4.6 × 100 mm, Agilent, CA, USA) at 30°C with a mobile phase composed of acetic acid (0.1%)-methanol (65:35) and a 0.8 ml/min flow rate. An external standard-based method was utilized for final quantitative analyses at 320 nm. Levels of FA in enzymatic hydrolysates were directly assessed *via* HPLC as above. All analyses were conducted in triplicate.

### Crude Enzyme Activity Analyses

Crude enzyme activity levels were assessed at 50°C in 50 mM sodium phosphate buffer (pH 5.0). Endoglucanase (CMCase) and xylanase activities were assayed in 2.5 ml reaction mixtures containing 100 μL crude enzymatic extract and carboxymethyl cellulose (CMC-Na) or beechwood xylan (Sigma, St. Louis, MO) at the final concentration of 1% or 0.2% (w/v) respectively. Reaction mixtures were incubated at 50°C for 30 min for CMCase or 10 min for xylanase, and the released reducing sugar was quantified by the Somogyi-Nelson method using glucose or xylose standard curves. Enzymatic activity on filter paper was determined in a similar method that was used to determine endoglucanase activity, by taking 30 mg of Whatman No. 1 filter paper in 2.5 ml of 50 mM sodium citrate buffer (pH 5.0) as the substrate. The activities of β-xylosidase, β-glucosidase, arabinofuranosidase, and glucuronidase were assessed in 1 ml reaction mixtures containing 900 μL of 50 mM sodium citrate buffer (pH 5.0), 50 μL enzyme solution, and respective 50 μL of 50 mM *p*-nitrophenyl β-D-xyloside, *p*-nitrophenyl β-D-glucoside, *p*-nitrophenyl α-L-arabinoside, or *p*-nitrophenyl β-D-glucuronide (Sigma, St. Louis, MO) as the substrates. After incubating at 50°C for 30 min, the reaction was stopped by adding 4 ml of glycine buffer (0.4 M, pH 10.8), and the liberated *p*-nitrophenol was measured at 405 nm. One unit (U) of enzyme activity was defined as the amount of enzyme required to liberate 1 μmol *p*-nitrophenol from the corresponding substrates per millilitre per minute under the assay conditions. The AXE activity was determined spectrophotometrically at 50°C by measuring the increasing in A_354_ nm during the initial 1 min of the assay resulting from the release of 4-methylumbelliferone from 4-methylumbelliferyl acetate. Reaction mixtures consisted of 1,390 μL 50 mM sodium citrate buffer (pH 5.0), 100 μL of 10 mM 4-methylumbelliferyl acetate and 10 μL crude enzyme. One unit of enzyme activity (U) was defined as the quantity of enzyme required to release 1 μmol of 4-methylumbelliferone per minute. FAE activity was assessed based upon the rate of methyl-ferulate (MFA) conversion into FA. Briefly, 100 μL of crude enzyme was mixed with 900 μL of 50 mM sodium citrate buffer (pH 5.0) containing 5.0 mM MFA. After incubating at 50°C for 30 min, the reaction was terminated at 99°C for 10 min. The released free FA was analysed using HPLC. One unit (U) of FAE activity was defined as the amount of enzyme liberating 1 μmol of free FA per min under the standard assay conditions. Benzyl alcohol release from benzyl-D-glucuronate was measured *via* HPLC to assess glucuronoyl esterase activity levels, which was similar to the determination of FAE activity. All assays were conducted in triplicate and the results were averaged together for final report.

### Date Analysis

One way analysis of variance (ANOVA) was conducted by SPSS software (Version 19.0) at *p* < 0.05 probability level. Multiple comparisons were conducted by Duncan method.

## Results and Discussion

### Wheat Bran Sample Composition

DSWB used in the present study was primarily composed of cellulose, xylan, and arabinan, accounting for 19.1, 29.2, and 22.2% of the total weight, respectively, demonstrating the high levels of carbohydrates therein (Table 1). Esterified FA and acetic acid levels in DSWB were 1.0 and 0.5%, respectively. The high FA and polysaccharide levels within this wheat bran input underscore the promise of DSWB as a source of fermentable monosaccharides and natural FA. Following autohydrolysis treatment (30 min at 160°C), marked arabinan depolymerization had occurred such that it accounted for just 13.1% of WBAR. Moreover, xylan content declined from 29.2 to 25.2%, indicating that arabinan and xylan exhibit different levels of autohydrolysis sensitivity. These findings are consistent with prior studies (Silva-Fernandes et al., 2015). Other component levels were also reduced following autohydrolysis with the exception of cellulose, the levels of which rose from 19.1 to 35.1%, suggesting that autohydrolysis did not significantly promote cellulose dissolution (Jiang et al., 2018; Tang et al., 2021). Additionally, it was found that there were also some acid insoluble constituents in both DSWB and WBAR, respectively accounting for 11.7 and 13.5%. These acid insoluble constituents might include lignin and ashes, etc.

**TABLE 1:** The content of different components in de-starched wheat bran and wheat bran autohydrolysis residue.

	DSWB^a^ (%)	WBAR^b^ (%)
Arabinan	22.2	13.1
Xylan	29.2	25.2
Mannan	0.6	0.3
Galactan	1.4	0.7
Cellulose	19.1	35.1
Glucuronic acid	5.0	2.6
Esterified ferulic acid	1.0	0.8
Acetic acid	0.5	0.3
Acid insoluble constituents	11.7	13.5

a DSWB, de-starched wheat bran.

b WBAR, wheat bran autohydrolysis residue.

### The Impact of Commercial Enzymatic Preparations on the Release of FA and Monosaccharides From WBAR

**FIGURE 1:**
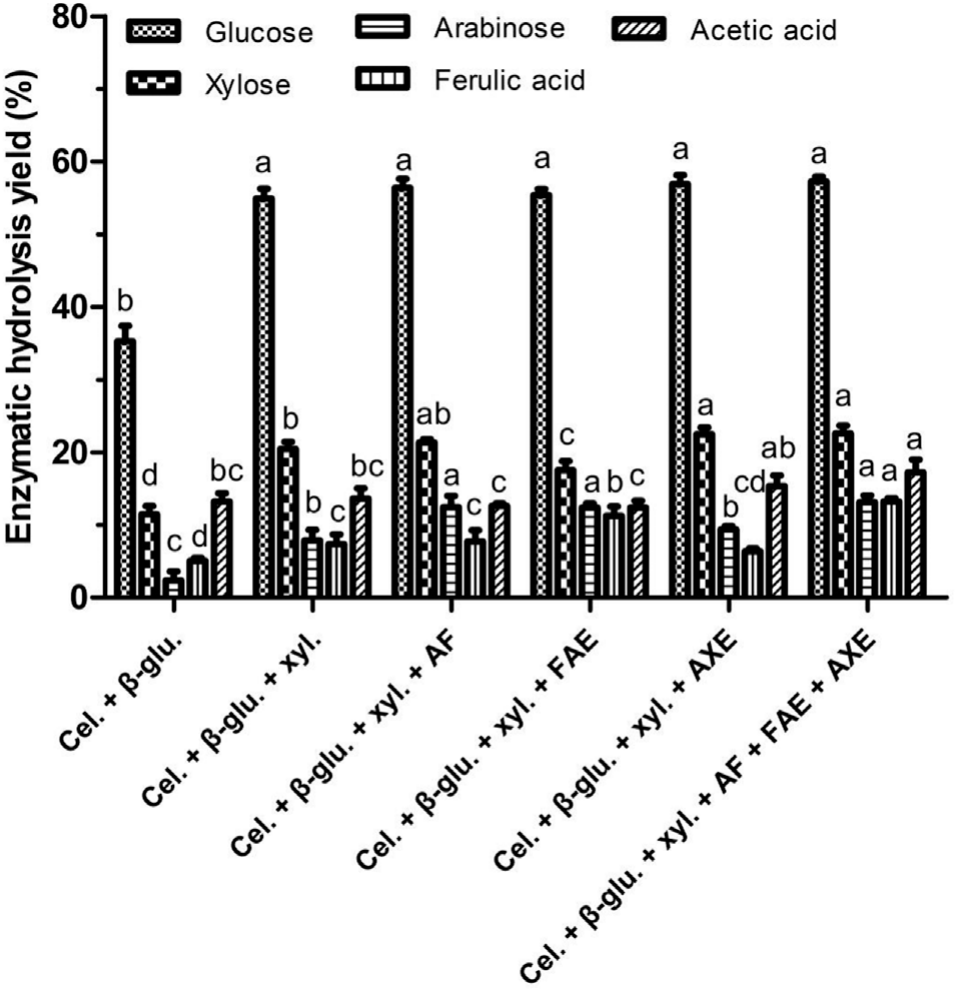
Monosaccharides and ferulic acid release from wheat bran autohydrolysis residue by commercial enzyme mixtures. Cel., cellulase; β-glu., β-glucosidase; Xyl., xylanase; AF, arabinofuranosidase; FAE, ferulic acid esterase; AXE, acetyl xylan esterase. Enzymatic hydrolysis yield of the same product between different treatments were conducted for one way analysis of variance (ANOVA) and multiple comparisons, where different lowercase letters indicate significant difference at *p* < 0.05 probability level.

Next, the impacts of the debranching enzymes FAE, AXE, and AF on the release of FA, monosaccharide, and acetic acid release from WBAR were assessed. As illustrated in Figure 1, the addition of both cellulase and β-glucosidase leaded to a glucose yield of 35.27% and xylose yield of 11.47% as the commercial cellulase also showed some xylanase activities. The addition of xylanase to cellulase and β-glucosidase resulted in respective increases in glucose and xylose yields by 55.68 and 78.47%, although overall xylose yield remained relatively low (20.47%). Adding AF to this enzymatic mixture increased arabinose yield by 57.92% to a relatively low final yield of 12.46%, while FAE addition improved FA yield by 52.17% to a final yield of 11.23%. Adding AXE to the enzymatic mixture did not significantly increase acetic acid yield. Finally, the combined addition of AF, FAE, and AXE as accessory enzymes resulted in respective xylose, arabinose, FA, and acetic acid yields of 22.58, 13.12, 13.25, and 17.22%. The heterogeneous makeup of wheat bran-derived arabinoxylan thus makes it difficult for these combinations of debranching enzymes to effectively degrade, with hydrolysis likely being restricted by steric hindrance or an absence of other enzymes including glucuronidase, β-xylosidase, glucuronoyl esterase, etc, which can catalyze the hydrolysis of certain atypical bond types including glucuronide linkages, glycosidic bonds, glucuronoyl esters, etc (Biely et al., 2016). A range of enzymes with different activity profiles is essential to enhance monosaccharide and FA release from wheat bran.

### Assessment of the Synergistic Hydrolytic Activity of Crude Enzymes Derived From Two Fungal Species

**FIGURE 2:**
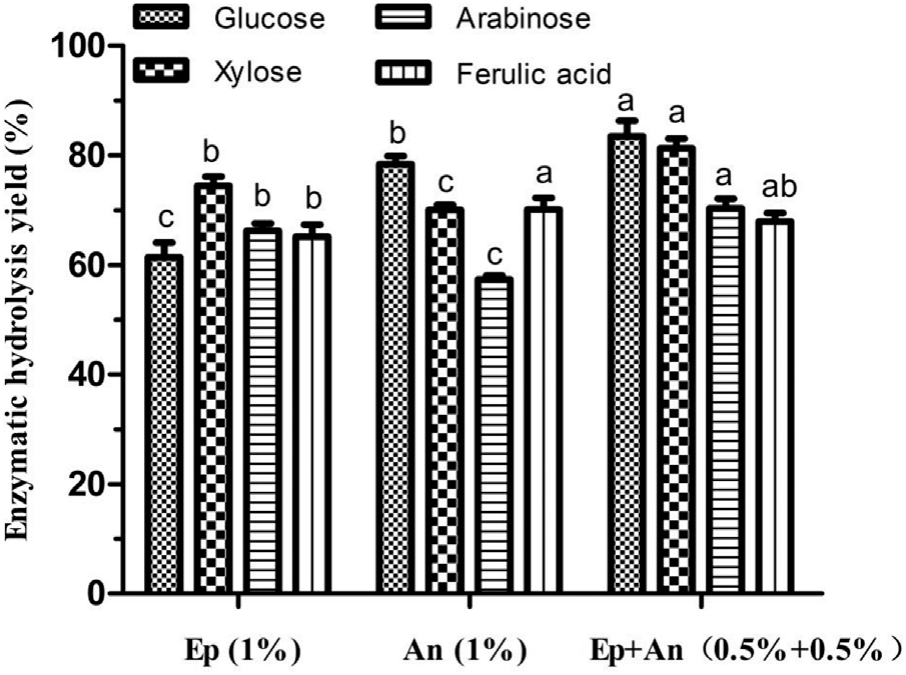
Effects of individual or blended enzyme on the enzymatic hydrolysis of wheat bran autohydrolysis residue. Ep, crude enzyme from *E. parvum*; An, crude enzyme from *A. niger.* Enzymatic hydrolysis yield of the same product between different treatments were conducted for one way analysis of variance (ANOVA) and multiple comparisons, where different lowercase letters indicate significant difference at *p* < 0.05 probability level.

As shown in Figure 2, the enzymatic yields of glucose, xylose, arabinose and ferulic acid were respectively 61.38, 74.39, 66.23 and 65.17% when using the crude enzyme of 1% (w/w) from *E. parvum*, while that were respectively 78.35, 70.02, 57.37 and 70.12% when using the same enzyme dose from *A. niger.* However, 83.46% of glucose, 81.28% of xylose, 70.34% of arabinose and 67.89% of ferulic acid were respectively yielded when using an equal-parts mixture of 0.5% (w/w) of each crude enzyme extract*.* Therefore, generally, an enzymatic blend derived from *A. niger* and *E. parvum*, which consisted of an equal-parts mixture of 0.5% (w/w) of each crude enzyme extract, achieved greater hydrolysis efficiency than did either enzyme crude individually at an equivalent dose (1%, w/w). Relative to an equivalent dose of *E. parvum*-derived enzymes, the enzymatic blend yielded glucose, xylose, and arabinose levels that were 35.97, 9.26, and 6.21% higher, respectively, with FA yield showing no significant difference. Similarly, relative to *A. niger*-derived enzyme yields, the enzymatic blend yielded glucose, xylose, and arabinose levels that were 6.52, 16.08, and 22.61% higher, while FA yield showed no significant difference either. Table 2 compiles the cellulolytic and hemicellulolytic activity profiles for these crude enzyme preparations from *A. niger* and *E. parvum*. *E. parvum* enzyme extracts exhibited higher levels of hemicellulase activity (including xylanase and arabinofuranosidase), whereas *A. niger* enzyme extracts exhibited more robust cellulase activity (including filter paper, CMC-Na, and β-glucosidase activity). The activities of xylanase and arabinofuranosidase from *E. parvum* respectively reached 53.4 U/mL and 76.8 U/mL, while that from *A. niger* were only 43.9 U/mL and 38.3 U/mL, respectively. On the other hand, the activities of filter paper, CMC-Na, and β-glucosidase from *E. parvum* reached 0.1 U/mL, 15.7 U/mL and 0.7 U/mL, whereas that from *A. niger* were significantly higher (0.2 U/mL, 46.3 U/mL and 3.8 U/mL, respectively). Both of these fungal enzyme preparations exhibited other accessory hydrolase activities for other side groups, including acetyl xylan esterase, FA esterase, and glucuronoyl esterase activity levels. The synergistically enhanced enzymatic yields of xylose and arabinose might be partly due to different glycoside hydrolase (GH) families which the related enzyme components were classified in the crude enzymes from *A. niger* and *E. parvum.* Such enhanced enzymatic hydrolysis appears to be attributable to the complementary activities of enzymes with different action modes and substrate specificities derived from these two fungi. Cooperative interactions between two or more hydrolytic components such that the product of one enzymatic reaction can serve as a substrate for another, produce combined total effects that are more than the sum of the effects of the components individually (E.M. Visser, et al., 2013; Saini et al., 2016). For example, α-L-arabinofuranosidases can be divided into GH 43 and GH 51. GH 51 shows enzyme activity on Xyl*p* (D-xylopyranosyl residue) monosubstituted by Ara*f* at either position 2 or 3, while GH 43 is specific for doubly arabinosylated Xyl*p* from which they selectively liberate only the α-1,3-linked Ara*f*, leaving the α-1,2-linked Ara*f* on the main chain (Sørensen et al., 2006; Sørensen et al., 2007b). As WB xylan is highly substituted with α-L-arabinofuranosyl residues singly attached to C(O)-2/C(O)-3, or doubly linked to both C(O)-2,3 of the xylose residues, the mutual action of these two groups of α-L-arabinofuranosidases can facilitate the synergistic debranching of all the α-L-arabinofuranosyls from xylan backbone and the complete enzymatic degradation of arabinoxylan into monosaccharides by the subsequent synergy action of endo-xylanase and β-xylosidase (Sørensen et al., 2006; Sørensen et al., 2007b). On the other hand, all the different groups of FA esterases can liberate *trans*-ferulic acid from 5-O-feruloylated Ara*f* (Biely et al., 2016). Furthermore, generally, *trans*-ferulic acid is terminally positioned on arabinofuranosyl in the short heterogeneous side chains or on the arabinofuranosyl moiety, and mainly exists in the form of 5-O-feruloylated Ara*f* (Saulnier et al., 1995; Agger et al., 2010). These might cause no significant synergistic enhancement of enzymatic ferulic acid yield using the enzyme blends as shown in Figure 2. Therefore, the crude enzymatic blend may thus be better suited to overall improvements in hydrolytic efficiency at a given enzyme dosage with concomitant reductions in costs.

**TABLE 2:** Activity profiles of the crude enzymes from solid state fermentation by *E. parvum* and *A. oryzae*. The activities of crude enzyme extracts from *E. parvum* and *A. niger* were conducted for one way analysis of variance (ANOVA) and multiple comparisons, where different lowercase letters indicate significant difference at *p* < 0.05 probability level.

	Enzyme produced by *E. parvum*(U/mL)	Enzyme produced by *A. niger*(U/mL)
Filter paper activity	0.1 ± 0.01b	0.2 ± 0.01a
CMC-Na^a^ activity	15.7 ± 0.7b	46.3 ± 0.7a
β-glucosidase	0.7 ± 0.04b	3.8 ± 0.02a
Xylanase	53.4 ± 1.2a	43.9 ± 1.6b
β-xylosidase	5.8 ± 0.7a	4.7 ± 0.3a
Acetyl xylan esterase	2.8 ± 0.02b	3.0 ± 0.01a
Arabinofuranosidase	76.8 ± 1.4a	38.3 ± 1.7b
Ferulic acid esterase	0.2 ± 0.01b	0.7 ± 0.1a
Glucuronoyl esterase	0.5 ± 0.02a	0.3 ± 0.02b
Glucuronidase	0.1 ± 0.01a	0.02 ± 0.01b

a CMC-Na, sodium carboxymethyl cellulose.

### The Effects of Different Enzyme Blends Dosages on Hydrolytic Efficiency

**FIGURE 3:**
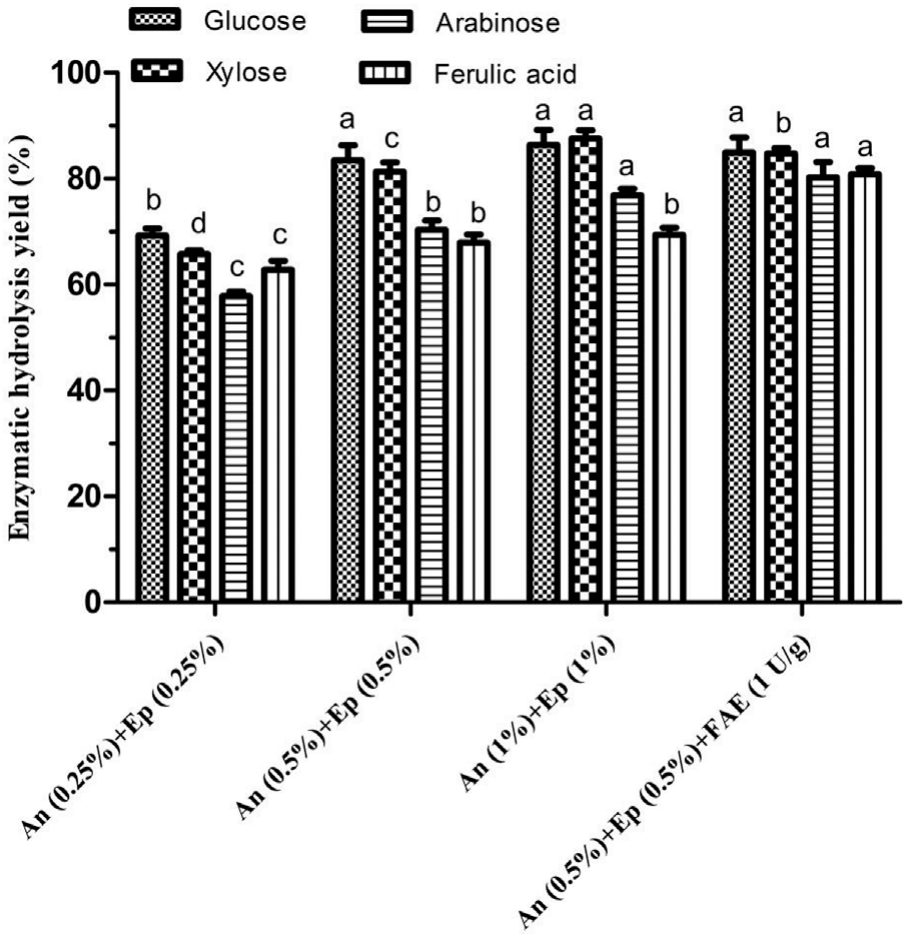
Effects of different blended enzyme dosages on the enzymatic hydrolysis of wheat bran autohydrolysis residue. Ep, crude enzyme from *E. parvum*; An, crude enzyme from *A. niger*; FAE, ferulic acid esterase. Enzymatic hydrolysis yield of the same product between different treatments were conducted for one way analysis of variance (ANOVA) and multiple comparisons, where different lowercase letters indicate significant difference at *p* < 0.05 probability level.

The effects of different *A. niger* and *E. parvum* enzyme blends (0.25–1%, w/w) on WBAR enzymatic hydrolysis were next assessed. Glucose, arabinose, xylose, and FA yields rose with increasing enzyme dosage (Figure 3). The yields of glucose, xylose, arabinose, and FA were respectively 69.26, 65.79, 57.82 and 62.78% with an addition of 0.25% enzyme dose. An enzyme dose of 0.5% was associated with glucose yield of 83.46%, xylose yield of 81.28%, arabinose yield of 70.34% and FA yield of 67.89%, achieving respective 20.50, 23.54, 21.65, and 8.14% improvements in glucose, xylose, arabinose, and FA yields relative to a 0.25% dose. However, these yields were respectively 86.37, 87.58, 76.89 and 69.37% when using 1% enzyme dose, with xylose and arabinose only respectively increased by 7.75 and 9.31%, yet glucose and FA yields displaying no significant difference relative to 0.5% dose. This suggested that the 0.5% dose was close to the saturation point. Besides, when additional FAE (1 U/g substrate) was added to this enzymatic mixture, FA and arabinose yields significantly rose by 19.10 and 14.07%, respectively. Arabinoxylan is heterogeneous and exhibits extensive covalent cross-linking between arabinofuranosyl and feruloyl residues. FAE was able to disrupt these linkages, thereby releasing arabinose and FA. Final respective glucose, xylose, arabinose, and FA yields were 84.98, 84.74, 80.24, and 80.86% of input. Reisinger et al. (Reisinger et al., 2013) previously reported glucose, xylose, and arabinose yields of 68, 55, and 48%, respectively, relative to raw wheat bran following hydrothermal treatment (30 min at 160°C) and enzymatic treatment with a mixture of 2% cellulase and 0.52% xylanase (w/w). Jiang et al. (Jiang and Guo, 2016) were only able to achieve respective glucose, xylose, and arabinose yields of 6, 12, and 1.5% when processing wheat bran *via* steam explosion (1.0 Mpa, 120 s) despite the application of high xylanase and cellulase doses (3% each, w/w). As such, these findings suggest that the enzymatic blend used in the present study can achieve hydrolytic outcomes superior to those in prior studies relying on commercial enzymes.

### Overall Mass Balance

**FIGURE 4:**
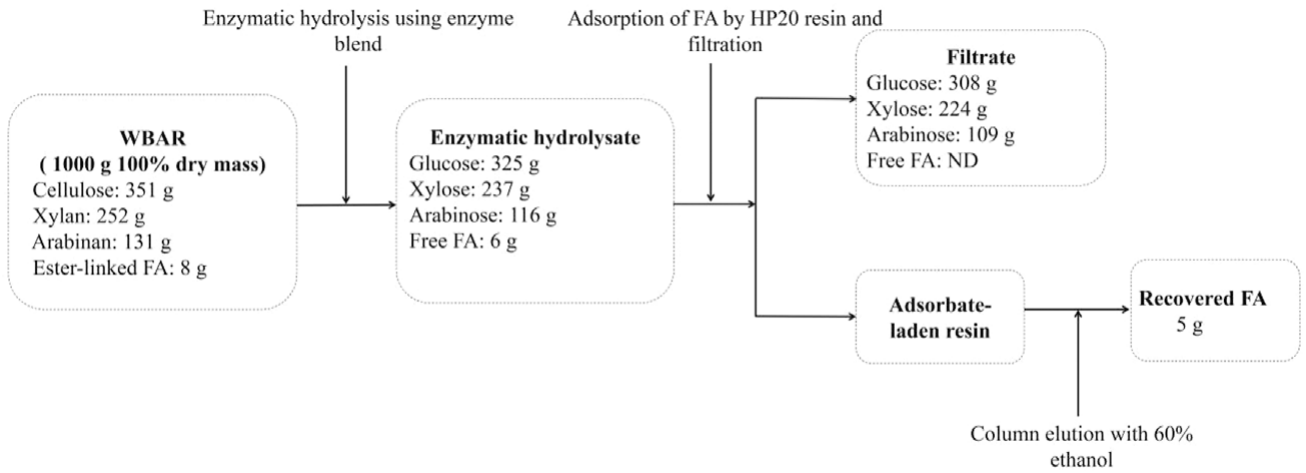
Mass balance of the monosaccharides and ferulic acid from wheat bran autohydrolysis residue. FA, ferulic acid; ND, not detected.

The overall composition of and mass balance for 1,000 g of dried WBAR over the course of the enzymatic hydrolysis process is outlined in Figure 4. Enzymatic hydrolysis was conducted at 50°C for 72 h using an enzymatic blend (0.5% derived from *A. niger* and 0.5% derived from *E. parvum* with an addition of 1 U/g FAE). This hydrolytic processing step yielded 325 g glucose, 237 g xylose, 116 g arabinose, and 6 g FA. Following the use of an HP-20 resin column for further FA enrichment, the final FA yield was 5 g, corresponding to 62.5% of the esterified FA present within the initial WBAR input. Levels of glucose, xylose, and arabinose in the resultant filtrate were 308 g, 224 g, and 109 g, respectively, corresponding to 79.0, 78.2, and 73.2% of the levels present within the initial WBAR input, with no FA remaining detectable within this filtrate. As such, FA can be reliably enriched from enzymatic hydrolysates through resin-based adsorption and subsequent ethanol elution.

## Conclusion

Herein, an enzyme blend derived from *A. niger* and *E. parvum* was used to achieve the enzymatic hydrolysis of wheat bran autohydrolysis residue, thereby achieving ferulic acid and monosaccharide co-production with high efficiency. Final ferulic acid recovery rates were as high as 62.5% of the levels of esterified ferulic acid present within the starting WBAR input, with limited monosaccharide loss following additional HP-20 resin-mediated separation. Together, these results suggest that wheat bran offers value as a biomass source that can be leveraged to prepare ferulic acid and to facilitate efficient enzymatic saccharification following autohydrolysis pretreatment.

## Data Availability

The raw data supporting the conclusions of this article will be made available by the authors, without undue reservation.
